# Spatial Variation of Bacterial Communities Associated With the Moss *Sanionia uncinata* on Deception Island, Antarctica

**DOI:** 10.1111/1758-2229.70467

**Published:** 2026-11-13

**Authors:** Kamila Barros Lima de Oliveira, Fabyano Alvares Cardoso Lopes, Stephan Machado Dohms, Marcelo Henrique Soller Ramada

**Affiliations:** ^1^ Programa de Pós‐Graduação em Ciências Genômicas e Biotecnologia Universidade Católica de Brasília Brasilia Brazil; ^2^ Laboratório de Microbiologia Universidade Federal do Tocantins Porto Nacional Brazil; ^3^ Curso de Medicina Veterinária Universidade Católica de Brasília Brasilia Brazil; ^4^ Programa de Pós‐Graduação em Gerontologia Universidade Católica de Brasília Brasilia Brazil

**Keywords:** community structure, extremophiles, metabarcoding, microbial diversity, moss, polar areas, polar volcano

## Abstract

Antarctic mosses play a fundamental ecological role in Maritime Antarctica, creating microhabitats that support diverse microbial communities. We used 16S rRNA gene (V3–V4) metabarcoding to investigate bacterial communities associated with the moss *Sanionia uncinata* at three sampling locations (seven sampling points, three samples each) within Antarctic Specially Protected Area No. 140 (Parts of Deception Island, South Shetland Islands). With sampling points as replicates, permutational multivariate analysis of variance (PERMANOVA; *R* ^2^ = 0.376, *p* = 0.0095) and analysis of similarities (ANOSIM; *R* = 0.73, *p* = 0.0095) based on Bray–Curtis dissimilarities showed that community composition differed among locations. Multivariate dispersion did not differ among locations, so these differences reflected changes in composition rather than within‐location variability. Alpha diversity did not differ among locations after correction for multiple comparisons, suggesting the moss microhabitat may buffer within‐sample diversity against external environmental variation. Functional profiles predicted with Phylogenetic Investigation of Communities by Reconstruction of Unobserved States (PICRUSt2) and Functional Annotation of Prokaryotic Taxa (FAPROTAX) were also similar among locations, which suggests functional redundancy. Our results indicate that *S. uncinata* harbours spatially structured bacterial communities with similar predicted functional capacities, and support the view that Antarctic mosses are important reservoirs of bacterial diversity.

## Graphical abstract

Bacterial communities associated with the Antarctic moss *Sanionia uncinata* differed in composition among three sampling locations on Deception Island, whereas alpha diversity and predicted functional profiles were similar. These results suggest that this moss harbours spatially structured bacterial communities with potential functional redundancy.

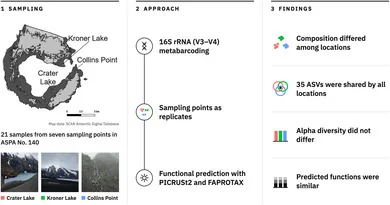

## 1 Introduction

Life in extreme environments has always intrigued scientists, and Antarctica hosts some of the most extreme environments on the planet, with high UV radiation, low temperatures, low nutrient availability and strong winds. Antarctica can be divided into Continental and Maritime Antarctica, with the maritime portion comprising the west of the peninsula and the nearby islands (Tatur 1989). Maritime Antarctica has a considerably milder and wetter climate than the continent, with mean summer monthly air temperatures above 0°C (Bañón et al. 2013; González‐Herrero et al. 2026), although low temperatures prevail for most of the year. Deception Island, located in the South Shetland Islands, stands out as a unique natural system within Maritime Antarctica due to its recent and continuous volcanic activity (Álvarez‐Valero et al. 2025). Furthermore, the island can serve as a natural model for studying biological colonization, ecological succession and adaptation to extreme environments (Convey et al. 2014).

As in the rest of Antarctica, the predominant macroflora on Deception Island is bryophytes, especially mosses (Ochyra et al. 2008). Despite the adverse conditions, Antarctic terrestrial ecosystems support adapted biological communities, in which mosses play a central role as major primary producers (Ochyra et al. 2008). Antarctic mosses, such as *Sanionia uncinata* , possess genetic controls and molecular mechanisms to alternate between growth and dormancy, responding to environmental stress and climatic variation (Yu et al. 2024). Furthermore, as one of the most abundant species in Antarctica, *S. uncinata* plays a fundamental role in structuring Antarctic terrestrial ecosystems, influencing the physical and chemical properties of the soil (Ochyra et al. 2008; Turetsky et al. 2012) and serving as an environment for the maintenance of diverse microbial communities and associated invertebrate communities (Convey and McInnes 2005; Câmara et al. 2021).

Bacterial communities associated with mosses are recognized as essential for plant fitness and environmental adaptation (Alvarenga and Rousk 2022). These organisms not only contribute to nutrient cycling and water retention, but also establish microenvironments that favour the colonization and maintenance of diverse microbial communities, establishing a symbiotic relationship with the moss (Bragina et al. 2013; Raymond 2016; Alvarenga and Rousk 2022). In extreme environments, these symbiotic associations can represent fundamental adaptive strategies for plant survival. There are studies on bacteria in the microbiome of Antarctic mosses in *S. uncinata* (Park et al. 2013; Câmara et al. 2021), and on polar fungi present in *S. uncinata* , *Bryum pseudotriquetrum* and *Polytrichastrum alpinum* (Zhang et al. 2013, 2021; Carvalho et al. 2020; Rosa et al. 2020), however, there is still a lack of information on the microbial composition in different regions of Antarctica. Additionally, whether bacterial community composition varies spatially across locations within the same island and whether such variation is driven by environmental filtering or spatial proximity remain largely unexplored.

Furthermore, ongoing climate change is altering temperature regimes and the availability of liquid water in Maritime Antarctica, and warming has been associated with increased moss growth on the Antarctic Peninsula (Amesbury et al. 2017). These changes may also affect moss‐associated microbial communities, potentially benefiting some taxa while threatening highly specialized endemic lineages adapted to current Antarctic conditions (Kleinteich et al. 2017; Holland‐Moritz et al. 2021).

To study these microorganisms, traditional culture‐based methods have provided limited information, as many Antarctic microorganisms are not cultivable under standard laboratory conditions (Rego et al. 2019). However, the study of these bacterial communities has been significantly expanded by the use of culture‐independent molecular techniques, such as 16S rRNA gene‐based metabarcoding. This approach allows for detailed characterization of bacterial diversity, including rare and uncultivable taxa, providing a comprehensive view of the structure of microbial communities in polar environments (Caporaso et al. 2012; Thompson et al. 2017).

In this context, the present study aims to investigate the structure of bacterial communities associated with the Antarctic moss *S. uncinata* on Deception Island, using 16S rRNA gene metabarcoding. We examine whether the spatial structuring of these communities is consistent with deterministic processes (e.g., environmental filtering), while acknowledging that environmental variables were not directly measured in this study. We seek to (i) evaluate the taxonomic composition and diversity of bacterial communities associated with the moss, (ii) compare alpha and beta diversity patterns among sampling locations and (iii) identify shared and location‐specific bacterial taxa. By focusing on a single moss species across multiple sampling locations on Deception Island, this study deepens our understanding of the composition and spatial organization of moss‐associated bacterial communities in Maritime Antarctica, using Deception Island as a model.

## 2 Materials and Methods

### 2.1 Study Area and Experimental Design

In February 2024, samples of *S. uncinata* were collected from three distinct sites within Antarctic Specially Protected Area (ASPA) No. 140 (Parts of Deception Island, South Shetland Islands; Figure 1A,B), namely Site A (Collins Point; Figure 1E), Site B (Crater Lake; Figure 1C) and Site K (Ronald Hill to Kroner Lake; Figure 1D), as designated in the management plan adopted by Measure 15 (2022) of the Antarctic Treaty Consultative Meeting. A total of 21 samples were collected across the three sites. At Crater Lake, nine samples were obtained from three sampling points located at 62°59′13.5″S, 60°39′57.1″W; 62°59′11.3″S, 60°40′00.9″W and 62°59′14.0″S, 60°39′54.3″W. At Kroner Lake, six samples were collected from two sampling points located at 62°58′46.0″S, 60°34′44.0″W and 62°58′40.8″S, 60°34′34.5″W. At Collins Point, six samples were obtained from two sampling points located at 62°59′58.1″S, 60°34′41.3″W and 62°59′58.1″S, 60°34′46.1″W. Three individual moss samples were collected at each sampling point.

**FIGURE 1:**
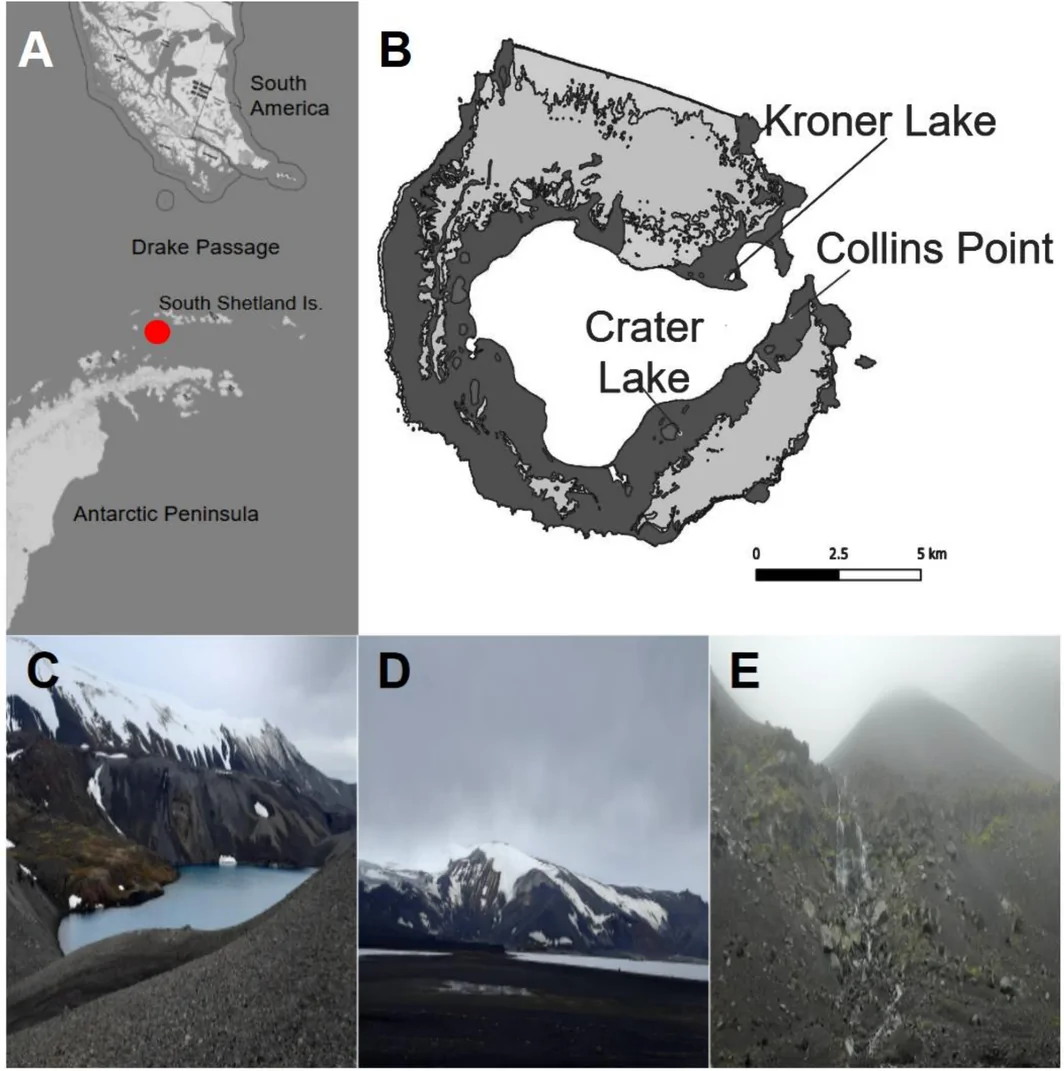
Geographic location of Deception Island and sampling locations. Map highlighting (red point) the location of Deception Island (A); map of Deception Island indicating sampling sites (B); overview of the three collection sites: Crater Lake (C); Kroner Lake (D) and Collins Point (E). Map data: (A) OpenStreetMap contributors; (B) data from the Scientific Committee on Antarctic Research (SCAR) Antarctic Digital Database, accessed 2026.

Sampling points were separated by at least 40 m (approximately 40–200 m apart) to reduce potential spatial dependence. Samples were collected in sterile bags and transported in an insulated bag with ice to a suitable location, where they were stored in a freezer at −80°C until processed in the laboratory.

### 2.2 Laboratory Processing and Sequencing

Total DNA from the samples was extracted in a laminar‐flow hood using the FastDNA Spin Kit for Soil (MP Biomedicals, Santa Ana, CA, USA), with modifications. Due to the rigidity of the material, mechanical lysis was performed using metal beads and the TissueLyser II (Qiagen) before extraction with the kit, and then the manufacturer's instructions were followed. The integrity of the material was verified on a 1% (w/v) agarose gel electrophoresis and quantified using the Qubit 2.0 Fluorometer (Invitrogen). The extraction protocol underwent several optimization rounds as initial extractions failed to yield detectable material, with only samples meeting minimum quality and quantity thresholds carried forward.

The DNA was stored in an Eppendorf tube and sent to BPI—Biotechnology Research and Innovation (Botucatu, São Paulo) for sequencing. The V3–V4 regions of the 16S rRNA gene were targeted for amplification. Metabarcoding sequences were obtained using the Illumina MiSeq platform (Illumina, San Diego, CA, USA). PCR amplification controls were performed by the sequencing facility, and libraries were subjected to quantification quality checkpoints before sequencing. The datasets produced in this study have been deposited in the NCBI Sequence Read Archive (SRA) and can be accessed through BioProject accession number PRJNA1478141.

### 2.3 Data From 16S rRNA Metabarcoding

Raw reads underwent quality control and adapter trimming using BBDuk v38.34 software (BBMap; Bushnell B., sourceforge.net/projects/bbmap). Parameters were used to exclude Illumina adapters and PhiX v3 control sequences; ktrim = l; k = 23; mink = 11; hdist = 1; minlen = 50; ‐‐tpe; ‐‐tbo; qtrim = rl; trimq = 20; ftm = 5; maq = 20.

Paired‐end reads were processed in QIIME2 v2024.10 (https://qiime2.org) (Bolyen et al. 2019). Noise removal, dereplication, merging of paired‐end reads and chimera removal were then performed using the DADA2 plugin (Callahan et al. 2016). The taxonomic classification of amplicon sequence variants (ASVs) was conducted using the qiime2‐feature‐classifier plugin (Bokulich et al. 2018), classify‐sklearn and the SILVA database version 138.1 (Quast et al. 2013). Additionally, sequences assigned to chloroplasts and mitochondria were removed, and rarefaction curves generated with the vegan package in R (version 4.5.1) were used to assess sequencing coverage. A per‐sample summary of read counts at each processing stage is provided in Supplementary Table S1.

Maps were prepared using QGIS version 3.44.3 (http://www.qgis.org). The overview map was generated using OpenStreetMap data, whereas the Deception Island map was produced using spatial layers from the Antarctic Digital Database (ADD) provided by the Scientific Committee on Antarctic Research (SCAR) (https://add.scar.org/). The numbers of ASVs that were shared between areas or exclusive to any of them were graphically represented with Venn diagrams using the InteractiVenn (Heberle et al. 2015). Alpha diversity (ASV richness, Shannon and Simpson indices and Pielou's evenness) was calculated using the PAST 4.03 program (Hammer et al. 2001). The remaining analyses were performed in R. The kruskal.test function was used to test whether there was a statistically significant difference in diversity indices among the three sampled areas. To account for multiple comparisons across four alpha diversity indices, both Bonferroni correction (adjusted threshold *p* < 0.0125) and FDR (false discovery rate) correction (Benjamini–Hochberg method) were applied (Benjamini and Hochberg 1995).

Beta diversity analysis was calculated using the Bray–Curtis and Jaccard distance metrics. Bray–Curtis dissimilarities were calculated from relative read abundance data obtained with the vegan decostand function (method = ‘total’), whereas Jaccard distances were calculated from presence/absence data generated using decostand (method = ‘pa’). To generate statistical support for observed differences, permutational multivariate analysis of variance (PERMANOVA; adonis2) and analysis of similarities (ANOSIM) were used to test differences among locations, with restricted permutations in which whole sampling points were permuted among locations while the three samples from each point were kept together (permute R package). With seven sampling points, all 5040 whole‐point permutations were enumerated (exact test; minimum attainable *p* = 0.0095). Variation among sampling points within locations was evaluated with a nested model (location/point), with permutations restricted within locations (9999 permutations). Non‐metric multidimensional scaling (NMDS) and a dendrogram were computed from Bray–Curtis dissimilarities to illustrate differences in bacterial composition among the three locations. All analyses were performed with the vegan (Oksanen et al. 2026) and permute (Simpson 2026) R packages, and *p*‐values below 0.05 were considered significant. Homogeneity of multivariate dispersion was assessed with betadisper, and differences in distances to location centroids were tested with the same whole‐point permutation scheme.

### 2.4 Prediction of Community Function

The functional prediction of the microbial community was performed using PICRUSt2 (Phylogenetic Investigation of Communities by Reconstruction of Unobserved States; Version 2.6.3), based on 16S rRNA gene sequencing data (Douglas et al. 2020). The MetaCyc database was used to infer functional profiles (Caspi et al. 2016). Pathways annotated in MetaCyc as engineered (i.e., artificial pathways constructed in genetically modified organisms and labelled ‘(engineered)’) were removed prior to all downstream analyses, as their prediction in environmental samples likely represents an artefact of the inference method (*n* = 5 pathways removed). The predicted pathway abundances were converted to relative abundance values for each sample. For heatmap visualization, the 30 most abundant pathways were selected based on mean relative abundance across samples.

The functional structure of the communities was evaluated by constructing a Bray–Curtis dissimilarity matrix based on abundances associated with metabolic pathways. To test differences between the sampled areas, PERMANOVA was performed using the adonis2 function from the vegan package in R, using the same whole‐point permutation scheme described in Section 2.3.

Additionally, taxonomy‐based functional inference was performed using the FAPROTAX (Functional Annotation of Prokaryotic Taxa) database (version 1.2.12) in Python (version 3.14.4), which associates prokaryotic taxonomic groups with predictive ecological functions (Louca et al. 2016). Low‐abundance functional groups (< 0.1%) were removed to reduce noise.

The data were standardized using z‐score transformation and used to construct heatmaps in R using the pheatmap package. The heatmaps were generated without hierarchical clustering to preserve the original ordering of samples.

## 3 Results

### 3.1 Assessment of Bacterial Diversity and Abundance

The total number of sequences was 62,333, distributed among 185 ASVs for the 21 samples analysed (103 at Crater Lake, 115 at Kroner Lake and 86 at Collins Point). Chloroplasts and mitochondria totalled 822,284 and 39,732, respectively, which were excluded from the analysis. The highest number of sequences was obtained in Crater Lake (28,350), followed by Kroner Lake (26,229) and Collins Point (7754). Rarefaction curves indicated that sequencing depth was sufficient to capture the bacterial diversity. Per‐sample read counts at each processing stage are provided in Supplementary Table S1. Because Collins Point yielded fewer bacterial reads, relative read abundance estimates for this location are based on lower coverage and should be interpreted with caution.

Pseudomonadota had the highest relative read abundance (35.70%), followed by Actinomycetota (26.01%), Acidobacteriota (15.09%), Bacteroidota (6.48%), Patescibacteria (4.78%), Myxococcota (3.87%), Chloroflexota (2.01%), Gemmatimonadota (1.55%), Verrucomicrobiota (1.31%), Cyanobacteria (1.11%) and Planctomycetota (1.10%). Abditibacteriota, Armatimonadota, Bdellovibrionota, Bacillota, Deinococcota, Dependentiae, Desulfobacterota, Elusimicrobiota and WPS‐2 represented less than 1% of the total reads. Although this proportion varied by sampling location, Pseudomonadota and Actinomycetota had the highest relative read abundances at all three sampling locations (Figure 2A).

**FIGURE 2:**
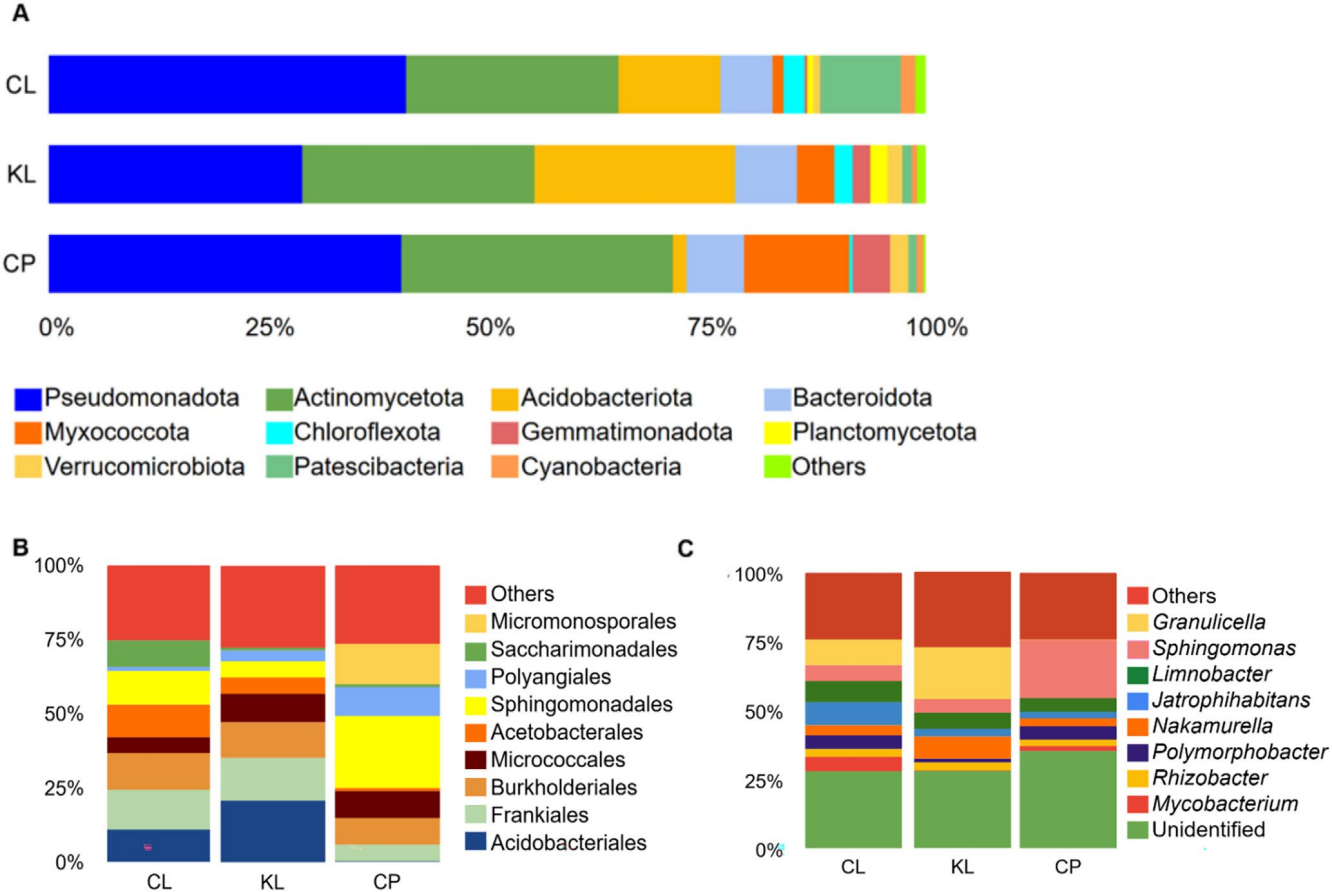
Relative read abundance of bacterial taxa in *Sanionia uncinata* sampled from three sampling locations on Deception Island, Antarctica (CL: Crater Lake; KL: Kroner Lake; CP: Collins Point). Taxonomic composition is shown at different hierarchical levels: (A) phylum, (B) order and (C) genus. Relative abundances are expressed as percentages of the total number of reads per location. Only the most abundant taxa per location are displayed, while low‐abundance groups are grouped under ‘Others’.

The most abundant order was Acidobacteriales (13.45%), followed by Frankiales (12.66%), Burkholderiales (11.69%), Sphingomonadales (10.31%), Acetobacterales (7.33%), Micrococcales (7.33%), Saccharimonadales (4.61%), Sphingobacteriales (3.82%), Polyangiales (3.28%), Corynebacteriales (2.87%), Xanthomonadales (2.63%), Ktedonobacterales (1.94%), Rhizobiales (1.76%), Micromonosporales (1.59%), Gemmatimonadales (1.54%), Chitinophagales (1.49%), Caulobacterales (1.45%), Solibacterales (1.30%), Cytophagales (1.22%), Cyanobacteriales (1.08%) and the other orders had less than 1% relative read abundance (Figure 2B). Unknown genera (27.72%) had the highest relative read abundance. *Granulicella* (11.57%), *Sphingomonas* (6.37%), *Limnobacter* (6.21%), *Jatrophihabitans* (5.22%), *Nakamurella* (4.99%), *Polymorphobacter* (3.21%), *Rhizobacter* (2.71%) and *Mycobacterium* (2.65%) were among the other abundant genera found (Figure 2C).

The three sampling locations shared 35 ASVs (Figure 3), belonging to the following taxonomic groups: *Acidiphilium*, *Allorhizobium‐Neorhizobium‐Pararhizobium‐Rhizobium*, BIrii41, *Candidatus* Solibacter, *Chthoniobacter*, *Clostridium*, env.OPS_17, *Ferruginibacter*, *Granulicella*, *Hymenobacter*, *Jatrophihabitans*, *Limnobacter*, LWQ8, *Methylobacterium‐Methylorubrum*, *Mucilaginibacter*, *Mycobacterium, Nakamurella*, *Nocardia*, *Nostoc*_PCC‐73102, *Pajaroellobacter*, *Polymorphobacter*, *Rhizobacter*, *Rhodanobacter*, *Sphingomonas*, WD260 and two ASVs assigned to the genus *Gemmatimonas*. The remaining eight ASVs could not be assigned to a known genus, but two belonged to the order Micrococcales and the others belonged to Frankiales, Burkholderiales, Caulobacterales, Rhizobiales, Saccharimonadales and Micropepsales.

**FIGURE 3:**
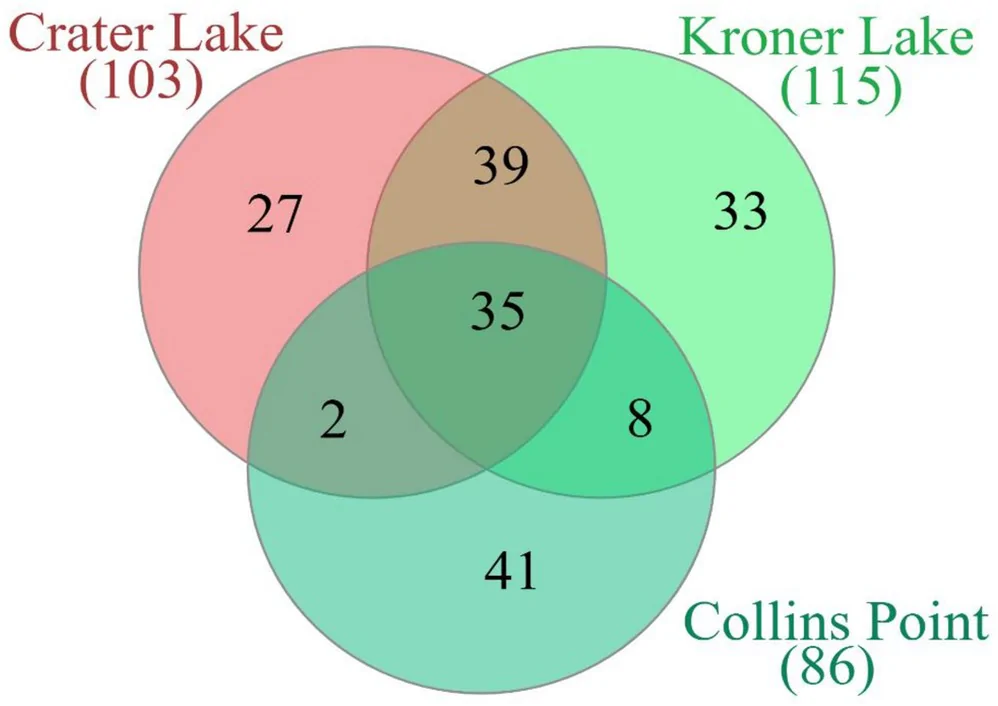
Venn diagram of bacteria in different areas of Deception Island. The total number of ASVs detected at each location is indicated in parentheses, while the values in exclusive regions indicate ASVs found uniquely in each location, and values at intersections represent ASVs shared between two or three areas.

### 3.2 Alpha and Beta Diversity

Before correction for multiple comparisons, Shannon (H′) (Figure 4C) and Simpson (1–D) (Figure 4D) diversity indices showed significant differences among areas (KruskalWallis test, *p* < 0.05), with Collins Point presenting comparatively lower values (Figure 4C–D). In contrast, Pielou's evenness showed no significant differences among areas (Figure 4B), while all samples exhibited relatively high evenness in the distribution of reads among taxa. Total taxonomic richness varied among sites, with Kroner Lake showing the highest value (115), followed by Crater Lake (103) and Collins Point (86). Pooled richness values were reported as descriptive measures, as they could be influenced by differences in sampling effort and sequencing depth. Overall, differences in richness among the three areas were not statistically significant (Figure 4A). After Bonferroni correction for four simultaneous tests (adjusted significance threshold, *p* < 0.0125), neither Shannon nor Simpson remained statistically significant. The Benjamini–Hochberg FDR procedure yielded the same conclusion: the *p*‐values for Simpson (0.01743), Shannon (0.04227), Pielou's evenness (0.07935) and richness (0.2235) did not meet their respective Benjamini–Hochberg critical values of 0.0125, 0.025, 0.0375 and 0.05. Thus, despite the lower number of reads obtained for Collins Point, no significant differences in alpha diversity indices were detected among the three areas after correction for multiple comparisons.

**FIGURE 4:**
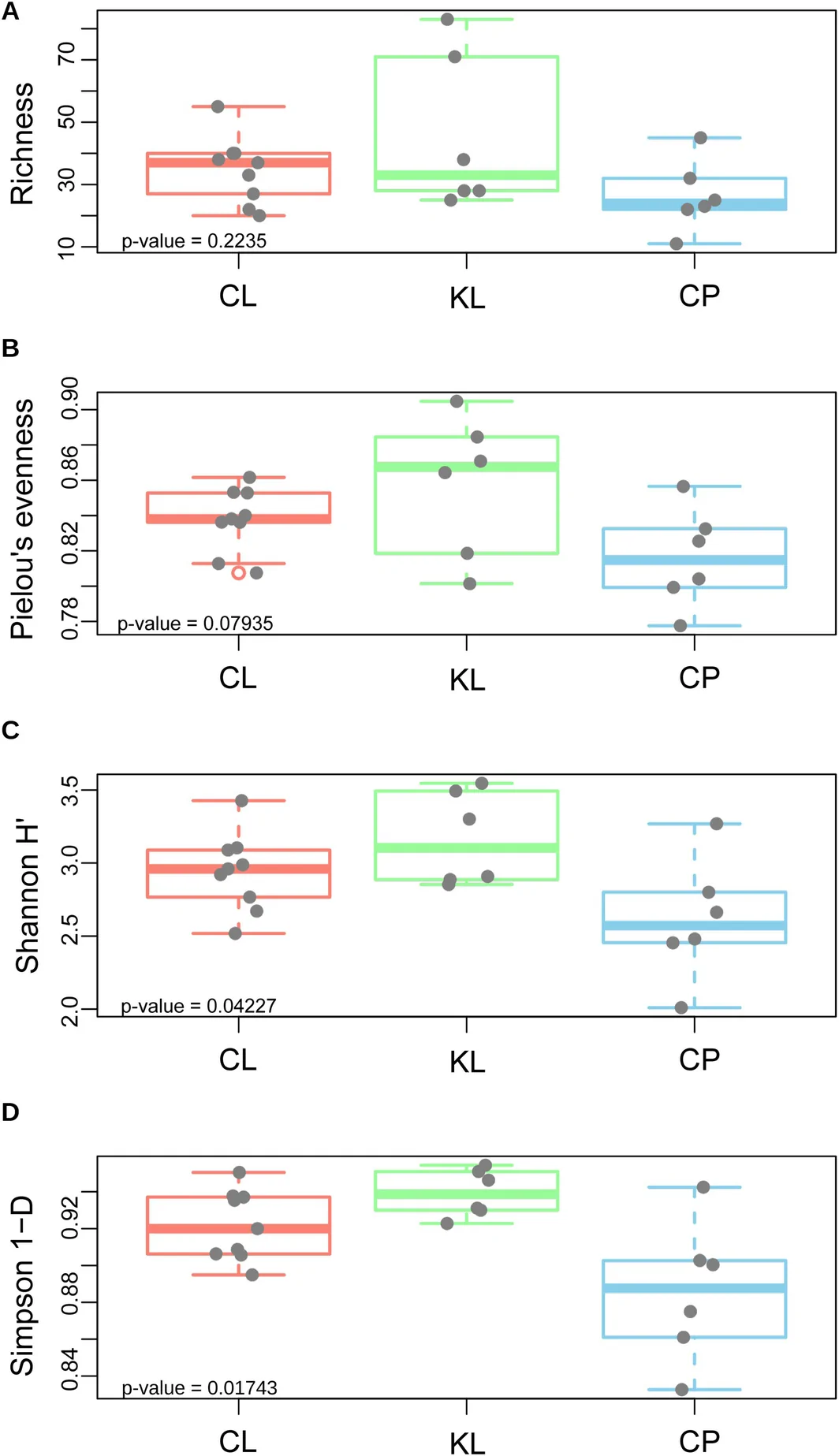
Alpha diversity estimators of bacterial communities of *Sanionia uncinata* from three locations (CL, Crater Lake; KL, Kroner Lake and CP, Collins Point). (A) Richness, (B) Pielou's evenness, (C) Shannon and (D) Simpson. Values shown are unadjusted Kruskal‐Wallis *p*‐values. After Bonferroni correction for four tests (threshold *p* < 0.0125) and Benjamini–Hochberg correction, none of the indices differed significantly among locations.

Analysis of bacterial community composition based on Bray–Curtis dissimilarities and visualized by non‐metric multidimensional scaling (NMDS) showed a reasonable stress level (0.14). The Bray–Curtis‐based NMDS showed a separation between the sampled areas (Figure 5A), and the dendrogram showed clustering by area (Figure 5B). Additionally, the samples from Kroner Lake were more similar to those from Crater Lake than to those from Collins Point (Figure 5B). PERMANOVA with whole‐point permutations indicated that community composition differed significantly among locations (*R* ^2^ = 0.376, *F* = 5.42, *p* = 0.0095), and ANOSIM yielded the same result (*R* = 0.733, *p* = 0.0095). In both tests, the observed grouping was the most extreme among all possible arrangements of sampling points. Sampling points within locations explained a further 24.1% of the variation (*R* ^2^ = 0.241, F = 2.21, *p* = 0.0025), indicating fine‐scale heterogeneity within locations.

**FIGURE 5:**
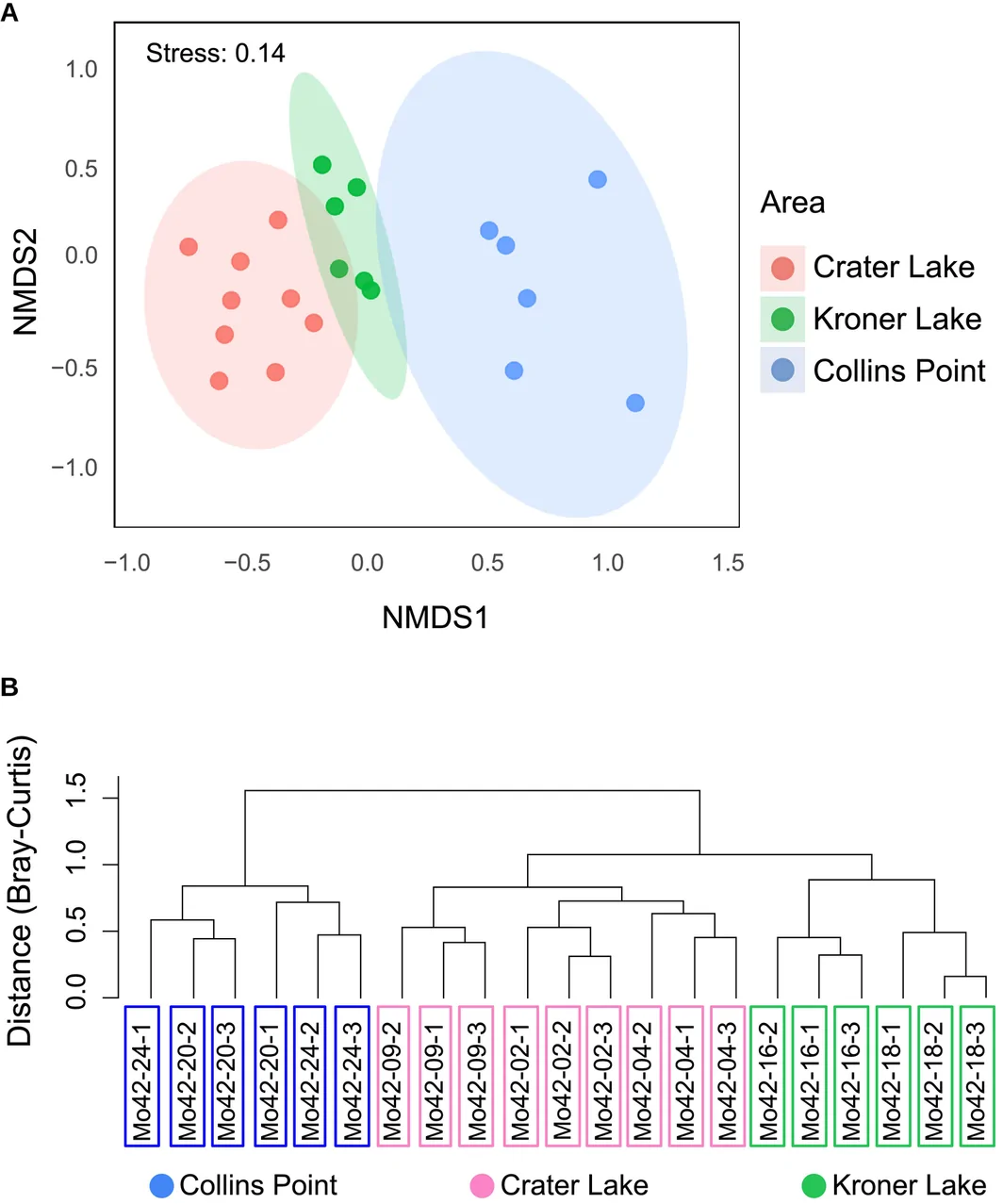
Community structure based on Bray–Curtis dissimilarity. (A) Non‐metric multidimensional scaling (NMDS) of three Deception Island areas: Crater Lake, Kroner Lake and Collins Point based on Bray–Curtis distance matrix, with ellipses representing the 95% confidence interval for a multivariate distribution and (B) dendrogram based on the Bray–Curtis distance matrix.

Multivariate dispersion did not differ significantly among locations (*F* = 1.02, *p* = 0.371), indicating that the differences detected by PERMANOVA reflected changes in community composition rather than greater heterogeneity within any one location.

Jaccard distances gave the same pattern. Location had a significant effect on community composition (PERMANOVA: *R* ^2^ = 0.243, *F* = 2.89, *p* = 0.0095; ANOSIM: *R* = 0.597, *p* = 0.0095), and sampling points within locations also differed (*R* ^2^ = 0.245, *F* = 1.67, *p* = 0.01).

Functional prediction analysis using PICRUSt2 and the MetaCyc database showed similar metabolic profiles across the sampled areas. The heatmap of the 30 most abundant metabolic pathways showed only subtle variations in pathway abundance among the samples, with most pathways exhibiting similar profiles across the three areas. The functional profiles predicted by PICRUSt2 were dominated by pathways related to energy metabolism, biosynthesis of essential compounds and degradation of organic substrates. Among the most representative pathways were those associated with fatty acid biosynthesis, amino acid metabolism and central pathways, including the pentose phosphate pathway and electron transfer processes (Figure 6A). No clear clustering pattern by sampling area was observed. PERMANOVA based on Bray–Curtis dissimilarity also did not indicate significant differences in the predicted functional structure between the sampled areas (*R* ^2^ = 0.168, *F* = 1.82, *p* = 0.333).

**FIGURE 6:**
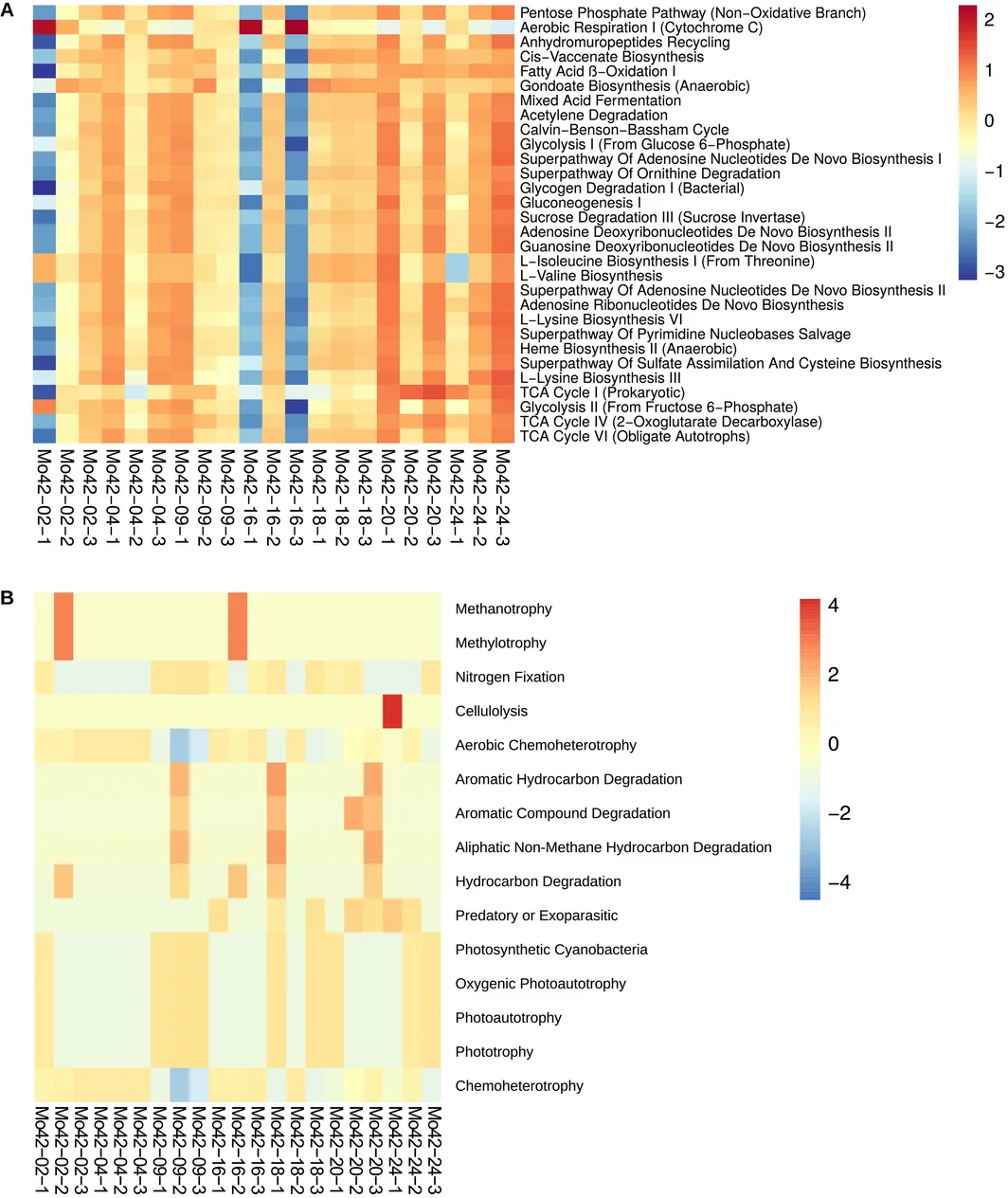
Microbial functional profiles inferred for *Sanionia uncinata* samples from Deception Island. Colours indicate relative variation in function among samples, ranging from low (blue) to high (red) abundance. (A) Heatmap of the 30 most abundant metabolic pathways predicted from 16S rRNA data using PICRUSt2 and annotated in the MetaCyc database. (B) Heatmap of ecological functions inferred from taxonomic classification using FAPROTAX.

The functional analysis based on FAPROTAX (Figure 6B) revealed consistent patterns, with a predominance of functions such as chemoheterotrophy, phototrophy and degradation of organic compounds. Functions related to the nitrogen cycle, such as nitrogen fixation, were also detected, but with no marked variation among the samples.

## 4 Discussion

### 4.1 Description of the Communities

Bryophytes are essential in Antarctic terrestrial ecosystems, participating in nutrient cycling, accumulating organic matter and buffering temperature fluctuations (Turetsky 2003; Parnikoza and Kozeretska 2020). These characteristics contribute to environmental stability in extreme conditions such as those found in Antarctica and can support complex microbial assemblages even under severe climatic stress (Zhang et al. 2025). Therefore, bacterial communities associated with mosses may differ significantly from those occurring in underlying soils, reflecting prolonged selection processes by plants and microenvironmental stabilization (Klarenberg et al. 2023; Ortiz‐Rivero et al. 2023).

This study characterized the bacterial communities associated with *S. uncinata* at three sampling locations on Deception Island (Crater Lake, Kroner Lake and Collins Point), an island formed by volcanic activity in Maritime Antarctica (Álvarez‐Valero et al. 2025). Although previous reports describe Antarctic soils as very diverse and microbiologically complex (Cary et al. 2010; Varliero et al. 2024; Dragone et al. 2025), there are few studies on the microorganisms associated with mosses (Park et al. 2013; Zhang et al. 2013, 2021; Carvalho et al. 2020; Rosa et al. 2020; Câmara et al. 2021), many of which have concentrated on fungal communities (Zhang et al. 2013, 2021; Carvalho et al. 2020; Rosa et al. 2020).

A substantial proportion of sequences assigned to chloroplasts and mitochondria were identified and removed from the dataset. This pattern is commonly reported in microbiome studies associated with bryophytes, as host organellar DNA is frequently co‐amplified by universal bacterial 16S rRNA primers, often representing a high fraction of the total reads (Câmara et al. 2021; Klarenberg et al. 2022, 2023). Although PCR amplification blanks were performed by the sequencing facility and only samples meeting minimum quality thresholds were carried forward, the possibility of low‐level reagent contamination cannot be entirely excluded, particularly given the low bacterial biomass and high proportion of host‐derived reads. The absence of extraction negative controls is a recognized limitation in low‐biomass microbiome studies (Eisenhofer et al. 2019), but is not exclusive to the present work, as other metabarcoding studies such as those of Bendia, Signori, et al. (2018), Câmara et al. (2021) and Ortiz‐Rivero et al. (2023) also did not report the use of extraction or sequencing negative controls. In addition, although all samples were sequenced in a single MiSeq run, DNA extractions were performed on different days, generally following the order of sample collection, and samples were not randomized across extraction days; an effect of processing day therefore cannot be fully separated from differences among locations. In any case, both issues should be addressed in future studies through the inclusion of negative controls and the randomization of samples across processing batches.

At all three locations, Pseudomonadota and Actinomycetota had the highest relative read abundances, together accounting for more than 50% of the reads. The predominance of these two phyla is consistent with studies of *S. uncinata* bacteria, although in those studies Bacteroidota was among the three most abundant phyla (Park et al. 2013; Câmara et al. 2021), a pattern not observed across all samples (Figure 2A). The dominance of Pseudomonadota and Actinomycetota in bacterial communities associated with other mosses has also been repeatedly observed in recent studies investigating moss microbiomes in both polar environments and other terrestrial systems (Klarenberg et al. 2023; Baev et al. 2024). Studies of moss microbiomes in Antarctica and cold regions indicate that Pseudomonadota and Actinomycetota are among the main groups in the bacterial communities inhabiting moss, frequently along with Acidobacteriota and Bacteroidota, reflecting important roles in the establishment of the moss microbiome (Klarenberg et al. 2023; Zhang et al. 2025). Furthermore, studies have reported that these bacterial communities remain structured by characteristics of the moss itself and not only by the soil matrix but also respond to the environment, mediating processes such as N fixation and carbon degradation/transformation (Klarenberg et al. 2022, 2023; Zhang et al. 2025).

The lower bacterial read counts at Collins Point reflected the higher proportion of chloroplast and mitochondrial reads removed from those samples (Supplementary Table S1), a pattern consistent with the known challenge of host DNA dominance in bryophyte‐associated microbiome studies (Câmara et al. 2021). While this uneven coverage may have influenced relative read abundance estimates shown in Figure 2A–C, the taxonomic profiles presented remained informative of the dominant bacterial lineages associated with *S. uncinata* at each location, as the most abundant phyla (Pseudomonadota and Actinomycetota) were consistently detected across all samples regardless of sequencing depth.

The dominance of Pseudomonadota may reflect its well‐documented metabolic versatility, tolerance to oligotrophic conditions and ability to colonize substrates disturbed or influenced by volcanic activity (Dawson et al. 2025). Members of this phylum are associated with nutrient cycling and stress tolerance in Antarctic soils (Weber and King 2017; Ortiz et al. 2020). Several members of Pseudomonadota harbour genes related to the oxidative stress response, heat shock proteins and versatile carbon metabolism pathways, including the degradation of aromatic compounds, which may confer adaptive advantages in geochemically heterogeneous substrates influenced by volcanic activity (Ayub et al. 2009; Tribelli et al. 2015; Musialowski et al. 2023). In cold ecosystems, representatives of Pseudomonadota, such as Burkholderiales and Sphingomonadales, have been associated with nitrogen transformations and organic matter cycling, contributing to nutrient cycling at the ecosystem level under low‐temperature conditions (Salazar et al. 2022; Quan et al. 2024). In the present study, the orders Burkholderiales and Sphingomonadales were the most abundant in the phylum Pseudomonadota (Figure 2B). Orders such as Burkholderiales include taxa capable of degrading complex organic compounds and tolerating oxidative and osmotic stress, which may be advantageous in Antarctic soils influenced by volcanic activity (Weber and King 2017).

Similarly, some Actinomycetota are known for their resilience to desiccation and freeze–thaw cycles, advantageous characteristics in polar terrestrial environments (Bradley et al. 2023; Tebele et al. 2025). Actinomycetes are also recognized for their ability to produce secondary metabolites, including antibiotics and enzymes active at low temperatures, which can enhance competitive interactions and survival under nutrient‐limited conditions (Barka et al. 2016). Frankiales was the most abundant order of Actinomycetota (Figure 2B).

Acidobacteriota, a well‐represented phylum in Crater Lake and Kroner Lake, mainly represented by Acidobacteriales and Solibacterales, was also highly abundant (Figure 2B). Although traditionally considered oligotrophic, Acidobacteriota is now recognized as metabolically diverse and well adapted to acidic and nutrient‐poor soils (Eichorst et al. 2018) under conditions of low pH, high metal concentration and edaphic stress (Kielak et al. 2016; Xu et al. 2022; Gonçalves et al. 2024), including Antarctic volcanic substrates.

Furthermore, the high proportion of unknown genera (27.72%) is noteworthy and consistent with previous Antarctic metabarcoding studies that reported a substantial fraction of unclassified bacterial diversity (Albanese et al. 2021; Cui et al. 2023). This pattern of microbial lineages detected by sequencing but lacking taxonomic classification or cultured representatives is called dark microbial matter and has also been reported in other extreme environments where a large proportion of microbial diversity remains unexplored (Li et al. 2023; Schultz et al. 2023; Han et al. 2024). An important limitation of metabarcoding studies in polar regions, often insufficiently acknowledged, is the incomplete coverage of reference databases for polar microbial diversity. Because Antarctic and sub‐Antarctic microbiomes remain severely underrepresented in these databases, a significant fraction of the detected ASVs cannot be assigned to known taxa, likely underestimating true diversity and biasing community composition estimates toward better‐characterized lineages. This limitation propagates into functional inference tools such as PICRUSt2 and FAPROTAX, whose predictions rely on reference genomes and taxonomy‐to‐function mappings (Louca et al. 2016; Douglas et al. 2020). This proportion of unidentified taxa emphasizes the limitations of conventional approaches and reinforces the value of integrating culture‐based methods with high‐resolution metagenomics and genomic analyses to improve the taxonomic and functional resolution of environmental microbiomes (Li et al. 2023; Schultz et al. 2023; Han et al. 2024).

It is important to highlight that the relative read abundances derived from 16S rRNA gene metabarcoding may not directly reflect bacterial cell abundances or biomass, as taxa with higher *rrn* operon copy numbers or greater complementarity to the primers used can generate disproportionately high read counts (Větrovský and Baldrian 2013). Correction for *rrn* copy number variation relies on reference databases such as rrnDB to assign copy numbers at the genus and species level (Klappenbach et al. 2001; Větrovský and Baldrian 2013). However, given that 27.72% of the bacterial reads in this study could not be assigned to a known genus, copy‐number correction would be unreliable for a substantial fraction of the dataset, as no reference copy numbers would be available for these unclassified lineages. Validation through alternative primer sets or shotgun metagenomics would therefore be needed to confirm whether the observed dominance of Pseudomonadota and Actinomycetota reflects true cell abundance. Nevertheless, the dominance of these phyla in moss‐associated bacterial communities has been consistently reported across Antarctic and sub‐Antarctic environments using both metabarcoding and culture‐dependent approaches (Park et al. 2013; Câmara et al. 2021).

Among the genera identified, *Granulicella*, *Sphingomonas*, *Limnobacter*, *Jatrophihabitans* and *Nakamurella* were consistently detected. These genera have been previously reported in Antarctic soils and cold environments and are frequently associated with polysaccharide degradation, organic acid metabolism and survival at low temperatures (Li et al. 2016; Pulschen et al. 2017; Coleine et al. 2019; Cui et al. 2023; Dragone et al. 2025).

Despite differences in relative read abundance patterns, the three locations shared 35 ASVs (Figure 3), representing a bacterial core dominated by genera such as *Granulicella*, *Sphingomonas*, *Limnobacter*, *Mucilaginibacter*, *Mycobacterium* and *Nakamurella*. The presence of a shared core microbiome in geographically separated locations suggests that environmental characteristics are an essential factor in community formation (Delgado‐Baquerizo et al. 2018; Peng et al. 2022) and that certain bacterial taxa are largely adapted to the environmental constraints imposed by Deception Island, including volcanic substrates and strong climatic stress. Furthermore, although Crater Lake and Kroner Lake are on opposite sides of the island (Figure 1B), they shared a greater number of ASVs than Kroner Lake and Collins Point, which are on the same side of the island (Figure 3). This pattern is consistent with a stronger role for environmental conditions than for spatial proximity in structuring these communities on Deception Island, although confirming this would require direct measurements of edaphic and physicochemical variables. Studies suggest a significant association between temperature, pH, salinity and chemical composition and the structure of the bacterial community on Deception Island (Bendia, Araujo, et al. 2018; Bendia, Signori, et al. 2018). It should also be noted that, given the sampling of three locations within a single island, the present study provides an initial characterization of spatial patterns in moss‐associated bacterial communities rather than a formal evaluation of isolation by distance, which would require broader sampling across greater geographic scales.

### 4.2 Alpha and Beta Diversity

In contrast to the observed differences in community composition among sites, alpha diversity did not differ significantly among locations after correction for multiple comparisons, regardless of the metric used. It is important to note that the number of bacterial reads varied considerably among locations (28,350 at Crater Lake, 26,229 at Kroner Lake and 7754 at Collins Point). As raw read counts were comparable across samples (Supplementary Table S1), this disparity mainly reflected the higher proportion of chloroplast and mitochondrial reads removed from the Collins Point samples (Section 4.1).

The absence of significant differences in alpha diversity across sites, despite clear compositional differences, may indicate that moss‐associated microbial communities maintain broadly similar levels of within‐sample diversity while undergoing shifts in taxonomic composition across contrasting environments. This pattern is consistent with the possibility that moss microhabitats provide relatively stable microscale conditions that partially buffer microorganisms from external environmental variation. However, this interpretation remains a hypothesis, as the present study did not directly assess the mechanisms underlying such buffering. Similar buffering effects have been reported in microbiomes associated with organisms from extreme environments (Klarenberg et al. 2023).

On the other hand, beta diversity analyses revealed clear and significant differences in community composition between sites. NMDS ordinations based on Bray–Curtis dissimilarities showed consistent separation of samples by sampling location, corroborated by PERMANOVA and ANOSIM results, indicating that spatial variation among areas accounted for approximately 37.6% of the observed variation in community composition. Notably, this result remained significant when whole sampling points, rather than individual moss samples, were treated as the units of replication, with both Bray–Curtis and Jaccard distances (PERMANOVA and ANOSIM, *p* = 0.0095 in all cases, the lowest value attainable with seven points), indicating that the location effect was not an artefact of pseudoreplication. Moreover, multivariate dispersion did not differ among locations, so these differences are unlikely to reflect greater heterogeneity within any single location. Nevertheless, with only two or three sampling points per location, inference about differences among locations rests on a small number of independent units, and broader sampling will be required to determine whether these patterns hold more widely on Deception Island. Sampling points within locations explained a further 24.1% of the variation, suggesting that fine‐scale microenvironmental heterogeneity may also contribute to structuring bacterial communities at a local scale (Bendia, Signori, et al. 2018).

The dendrogram based on the Bray–Curtis method revealed greater similarity between Kroner Lake and Crater Lake. Since the Bray–Curtis method incorporates abundance information, this pattern suggests that environmental conditions modulate dominance structures. The higher *R* ^2^ values obtained for the Bray–Curtis method compared to the Jaccard method reinforce the idea that the differences between the areas are primarily driven by differences in relative abundance rather than by taxon presence or absence. In other systems, environmental factors such as pH, moisture and substrate chemistry have been reported to influence microbial community structure more strongly than geographic distance (Delgado‐Baquerizo et al. 2018; Peng et al. 2022); whether the same holds on Deception Island remains to be tested with in situ environmental data.

Thus, these results highlight that Deception Island harbours diverse, structured and site‐specific bacterial communities, despite its extreme and volcanically active nature. Therefore, the coexistence of a shared bacterial core and site‐dependent relative read abundance patterns may indicate that Antarctic volcanic ecosystems are shaped by a combination of environmental filtering, microhabitat buffering and resilience mechanisms. As adjacent bulk soil was not sampled in this study, we cannot disentangle moss‐specific selection from the broader soil community; this remains an important direction for future work, together with the integration of metagenomic and functional analyses to elucidate the basis of the compositional differences between sites.

### 4.3 Functional Profiles of Communities

Although beta diversity analyses showed a clear separation of communities according to sampling location, and these differences could not be attributed to differences in within‐location dispersion, predicted functional profiles remained conserved. In the PICRUSt2 functional prediction analysis, the main metabolic pathways found were related to central carbon metabolism, amino acid biosynthesis and nucleotide metabolism, while FAPROTAX mainly identified functions associated with chemoheterotrophy and the carbon cycle.

Compared to PICRUSt2, FAPROTAX (Figure 6B) generated a more restricted functional profile, probably due to its conservative taxonomy‐based approach (Louca et al. 2016). A significant limitation of this study is the high proportion of unknown genera (27.72%), indicating that a substantial fraction of the microbiome remains unrepresented in available reference databases. Since PICRUSt2 relies on reference genomes to infer metabolic functions (Douglas et al. 2020), its accuracy may be reduced in poorly characterized environments, such as polar ecosystems. Still, in agreement with the patterns of the PICRUSt2 heatmap (Figure 6A), the PERMANOVA analysis did not detect significant differences in functional structure between the sampled areas, despite the clear taxonomic differentiation observed. This pattern is consistent with the concept of functional redundancy and suggests that core functional potential is maintained despite compositional differences among the sampling locations (Louca et al. 2018; Zhang et al. 2025).

Finally, it is important to emphasize that predictions based on 16S rRNA have limitations and some predicted functions should be interpreted with caution (Větrovský and Baldrian 2013; Douglas et al. 2020). This is particularly relevant for functions associated with microbial groups that were not detected in the taxonomic data. In polar ecosystems, this limitation may be further intensified by the underrepresentation of Antarctic taxa in reference databases (Louca et al. 2016; Douglas et al. 2020), as discussed above (Section 4.1).

## 5 Conclusions

This study characterized the bacterial communities associated with the moss *S. uncinata* at three sampling locations on Deception Island using 16S rRNA gene metabarcoding. Even under the extreme conditions of this volcanic island, all locations harboured diverse bacterial communities, with Pseudomonadota and Actinomycetota accounting for the highest relative read abundances. Alpha diversity was similar among locations, but community composition differed significantly, and this difference held under a conservative test that treated whole sampling points as the units of replication. Predicted functional profiles varied little among locations, which suggests functional redundancy within the moss‐associated microbiome.

These findings are relevant in three respects. Because the same moss species was sampled at every location, the compositional differences are not explained by differences in host, and they show that moss‐associated bacterial communities can be spatially structured within a single island, a scale that has received little attention in Antarctica. The combination of compositional differences and similar predicted functions also suggests that these communities may retain their metabolic potential as local conditions change, which is relevant for anticipating how they may respond to warming and to shifts in liquid water availability in Maritime Antarctica. Finally, the samples come from ASPA 140, an area with restricted access, and the sequences deposited in the NCBI Sequence Read Archive add to the still limited reference data available for polar bacteria.

Broader sampling, in situ environmental measurements and metagenomic approaches will help identify the factors that structure these communities. The present study provides a baseline for such work and for monitoring moss‐associated microbiomes on Deception Island as environmental change continues.

## Author Contributions

**Kamila Barros Lima de Oliveira:** conceptualization, writing – original draft, investigation, methodology, visualization. **Fabyano Alvares Cardoso Lopes:** methodology, visualization, investigation. **Stephan Machado Dohms:** investigation, methodology. **Marcelo Henrique Soller Ramada:** writing – review and editing, resources, project administration, conceptualization, funding acquisition, writing – original draft.

## Funding

This work was supported by Conselho Nacional de Desenvolvimento Científico e Tecnológico (442720/2018‐1, 440916/2023‐2) and Coordenação de Aperfeiçoamento de Pessoal de Nível Superior (88887.703211/2022‐00).

## Ethics Statement

The authors have nothing to report.

## Consent

The authors have nothing to report.

## Conflicts of Interest

The authors declare no conflicts of interest.

## Supporting information


**Supplementary Table S1:** Per‐sample summary of sequencing read counts at each processing stage. Raw: total reads obtained from sequencing; After BBDuk: reads remaining after adapter trimming and quality filtering with BBDuk; Denoised: reads retained after DADA2 denoising of forward and reverse reads; Merged: reads after paired‐end merging; Non‐chimeric: reads after chimera removal; Final bacterial reads: reads retained after removal of chloroplast and mitochondrial sequences.

## Data Availability

The data that support the findings of this study are openly available in NCBI Sequence Read Archive, BioProject accession number PRJNA1478141. All other data are presented herein and can be requested upon contact with the corresponding author.
