# Supplementary material for: Spatial Variation of Bacterial Communities Associated With the Moss Sanionia uncinata on Deception Island, Antarctica
Source: Environ Microbiol Rep. 2026 Nov 13;18(6):e70467. doi: 10.1111/1758-2229.70467 (PMC13768745; doi:10.1111/1758-2229.70467)
Supplement: Supplementary file 1 [file EMI4-18-e70467-s001.docx]

Supplementary Table S1. Per-sample summary of sequencing read counts at each processing stage. Raw: total reads obtained from sequencing; After BBDuk: reads remaining after adapter trimming and quality filtering with BBDuk; Denoised: reads retained after DADA2 denoising of forward and reverse reads; Merged: reads after paired-end merging; Non-chimeric: reads after chimera removal; Final bacterial reads: reads retained after removal of chloroplast and mitochondrial sequences.

| Sample | Sampling location | Raw | After BBDuk | Denoised | Merged | Non-chimeric | Final bacterial reads |
| --- | --- | --- | --- | --- | --- | --- | --- |
| Mo42-02-1 | Crater Lake | 34,805 | 30,577 | 29,306 | 23,330 | 23,250 | 5,951 |
| Mo42-02-2 | Crater Lake | 65,094 | 60,295 | 58,441 | 51,676 | 50,939 | 5,269 |
| Mo42-02-3 | Crater Lake | 54,880 | 51,587 | 49,966 | 45,380 | 44,747 | 2,396 |
| Mo42-04-1 | Crater Lake | 60,341 | 57,109 | 55,772 | 52,677 | 52,147 | 1,300 |
| Mo42-04-2 | Crater Lake | 56,892 | 53,366 | 51,195 | 46,492 | 46,053 | 3,463 |
| Mo42-04-3 | Crater Lake | 62,575 | 57,752 | 56,638 | 53,549 | 52,915 | 1,285 |
| Mo42-09-1 | Crater Lake | 58,110 | 54,934 | 53,915 | 51,044 | 50,767 | 1,153 |
| Mo42-09-2 | Crater Lake | 59,347 | 55,440 | 53,296 | 48,224 | 47,408 | 3,844 |
| Mo42-09-3 | Crater Lake | 53,563 | 49,932 | 48,267 | 43,887 | 42,721 | 3,689 |
| Mo42-16-1 | Kroner Lake | 56,868 | 53,693 | 50,855 | 44,015 | 43,382 | 8,571 |
| Mo42-16-2 | Kroner Lake | 52,825 | 50,161 | 48,432 | 44,857 | 44,394 | 1,843 |
| Mo42-16-3 | Kroner Lake | 58,136 | 54,736 | 52,234 | 45,384 | 44,338 | 9,513 |
| Mo42-18-1 | Kroner Lake | 53,956 | 51,296 | 49,885 | 45,869 | 45,203 | 2,298 |
| Mo42-18-2 | Kroner Lake | 50,393 | 48,018 | 46,402 | 42,737 | 41,813 | 2,020 |
| Mo42-18-3 | Kroner Lake | 49,015 | 46,479 | 44,761 | 40,528 | 39,987 | 1,984 |
| Mo42-20-1 | Collins Point | 52,862 | 50,325 | 48,810 | 45,000 | 44,728 | 679 |
| Mo42-20-2 | Collins Point | 52,848 | 50,001 | 47,547 | 43,320 | 43,036 | 2,502 |
| Mo42-20-3 | Collins Point | 61,118 | 58,095 | 56,142 | 52,488 | 51,847 | 1,198 |
| Mo42-24-1 | Collins Point | 31,460 | 29,493 | 26,849 | 22,931 | 22,769 | 1,903 |
| Mo42-24-2 | Collins Point | 49,938 | 47,010 | 45,708 | 43,821 | 43,692 | 1,105 |
| Mo42-24-3 | Collins Point | 54,209 | 51,176 | 50270 | 48572 | 48213 | 367 |
